# Supplementary material for: Peripheral blood transcriptome profiling enables monitoring disease progression in dystrophic mice and patients
Source: EMBO Mol Med. 2021 Mar 10;13(4):e13328. doi: 10.15252/emmm.202013328 (PMC8033515; doi:10.15252/emmm.202013328)
Supplement: Supplementary file 1 — Appendix [file EMMM-13-e13328-s005.pdf]

APPENDIX FIGURES

Table of Contents

- Legends for Appendix Figures S1-6
- Appendix Figures S1-6

Appendix Figure S1

A) Matrix with the values of the Jaccard Index between blood and muscle module eigengenes.
B) Matrix with the values of the overlap coefficient between blood and muscle module eigengenes.

Appendix Figure S2

Comparison of the logFCs in the PS49 and in the PMO groups to the desired drug effect.

Appendix Figure S3

Boxplots comparing the distribution of CSF1, TSP1, ADSL and EEF1B2 observed in this study to those obtained in Spitali et al. (2018). The central band of the boxplot denotes the median; the box denotes the first and third quartile; the whiskers are computed as min(max(x), Q3 + 1.5 IQR) and max(min(x), Q1 – 1.5 * IQR), where min(x) and max(x) denote the minimum and maximum of the distribution, Q1 and Q3 the first and third quartile, and IQR the interquartile range.

Appendix Figure S4

Boxplots with the distribution of the 18 genes that are significantly associated with steroid treatment. The central band of the boxplot denotes the median; the box denotes the first and third quartile; the whiskers are computed as min(max(x), Q3 + 1.5 IQR) and max(min(x), Q1 – 1.5 * IQR), where min(x) and max(x) denote the minimum and maximum of the distribution, Q1 and Q3 the first and third quartile, and IQR the interquartile range.

Appendix Figure S5

A) Correlogram showing the correlation between body measurements in the DMDMex dataset.

B) Circle of correlations for the principal component analysis of the body measurements in the DMDMex dataset.

C) Volcano plot for the F test on the association between gene expression and body measurements.

D, E, F, G) Scatter plots showing the relationship between the first principal component of the body measurements (x axis) and the expression of CREG1, MGST1, MRC2 and SP140 (y axis).

Appendix Figure S6

A) Correlogram showing the correlation between performance tests in the DMDMex dataset.

B) Volcano plot for the F test on the association between gene expression and performance tests.

C, D, E, F) Scatter plots showing the relationship between arm domain, activities of daily living domain, North Star Ambulatory Assessment and Brooke scale (x axis), and the expression of LAPTM4B (y axis).

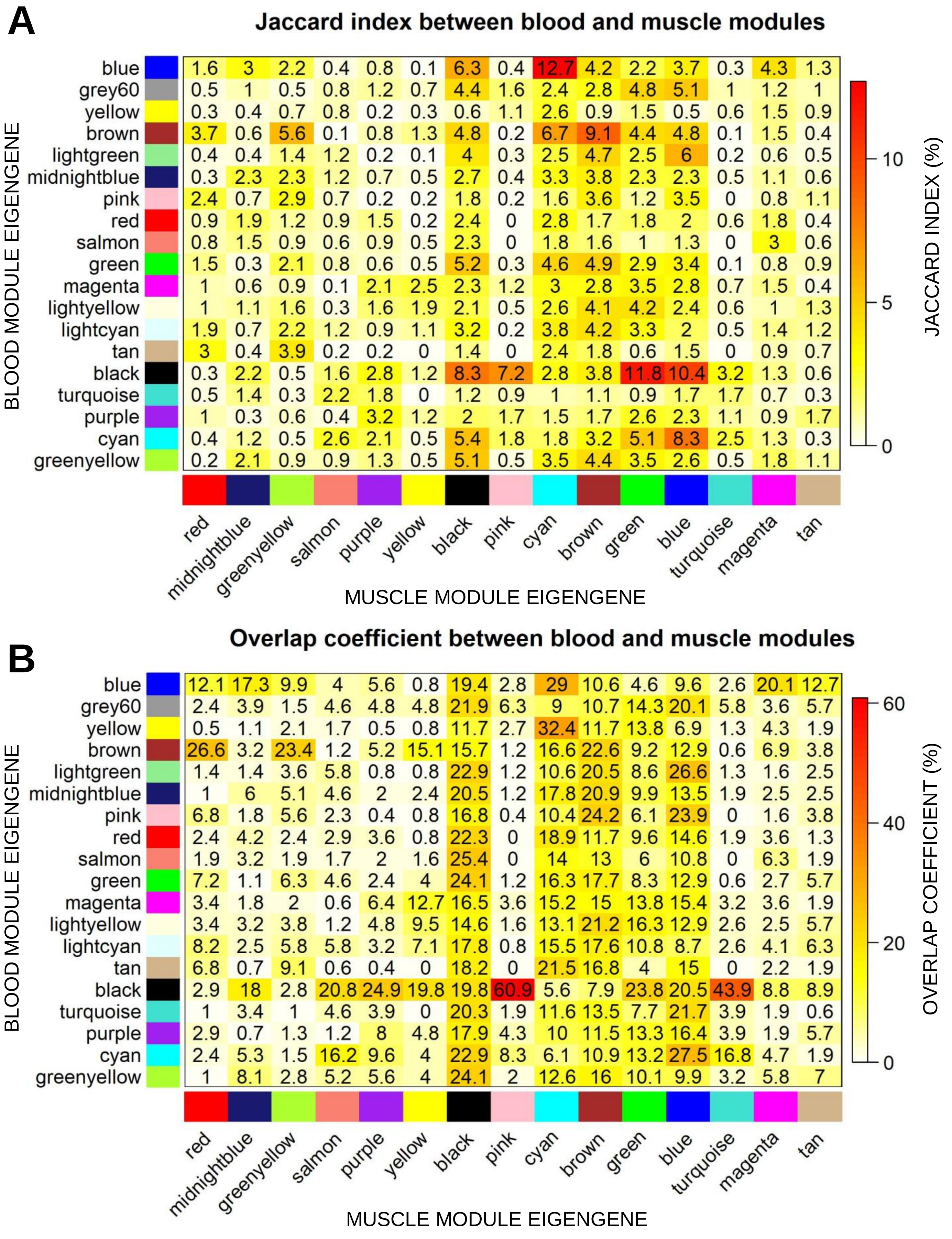

Appendix Figure S1

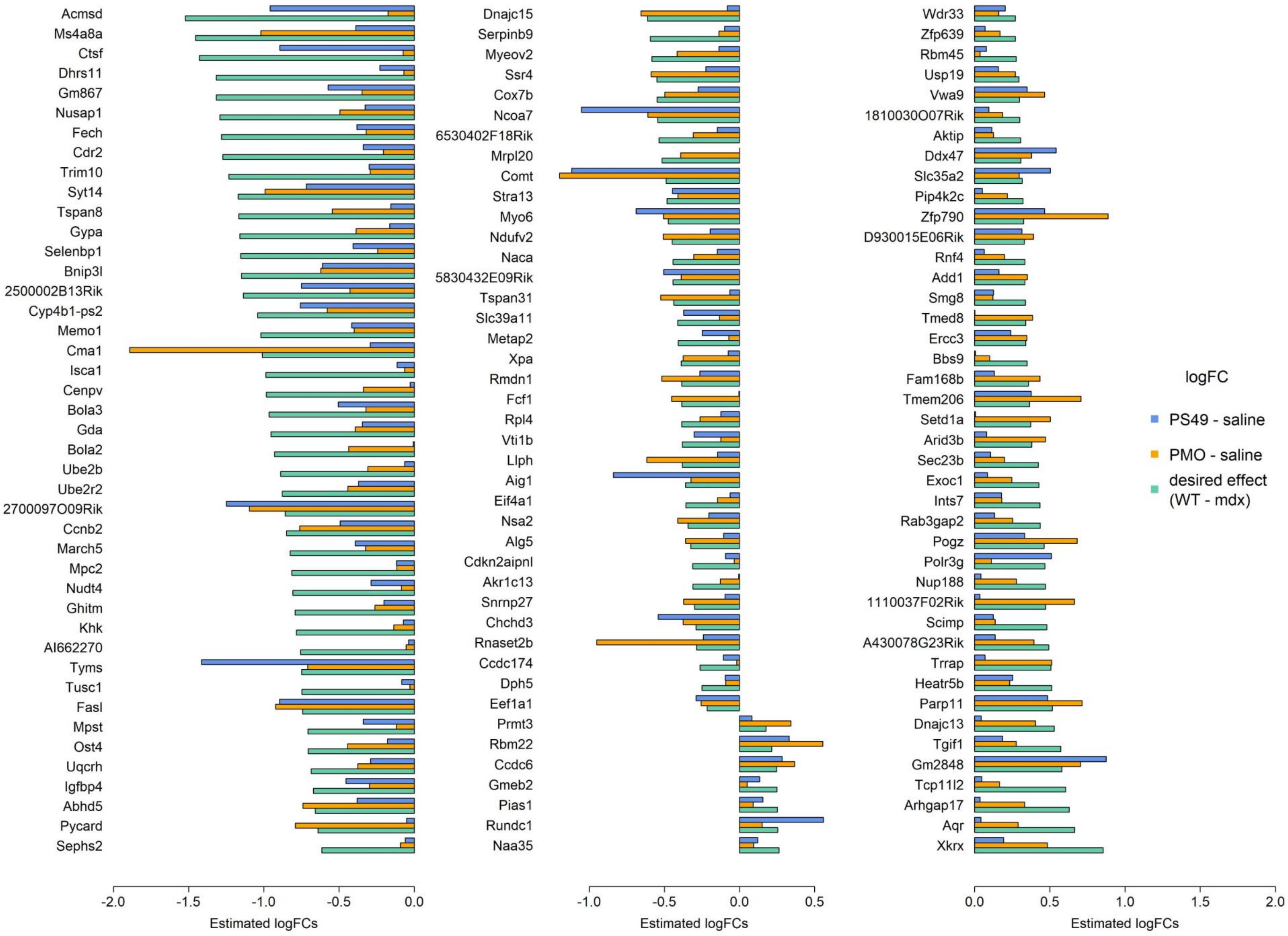

Appendix Figure S2

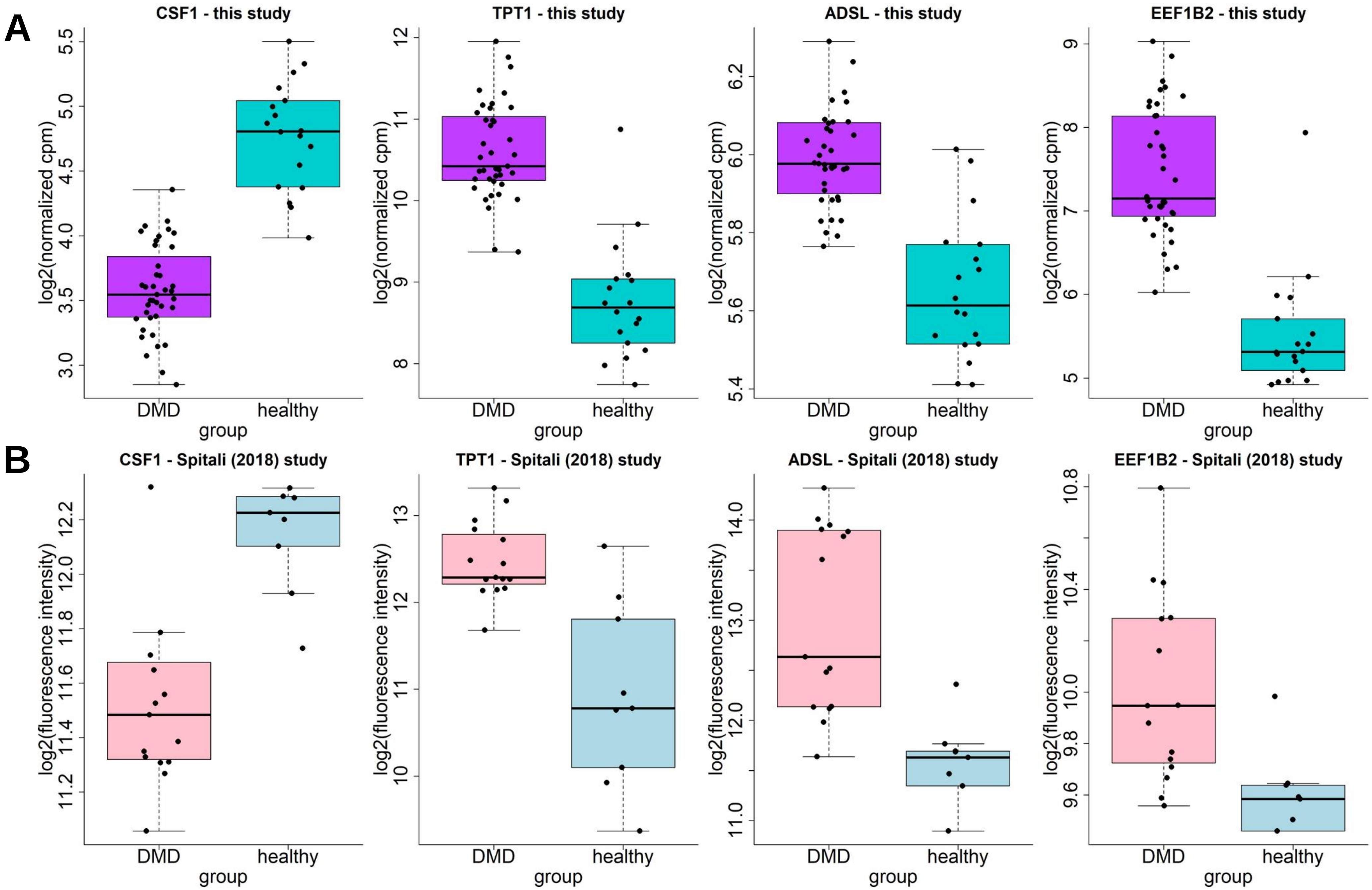

Appendix Figure S3

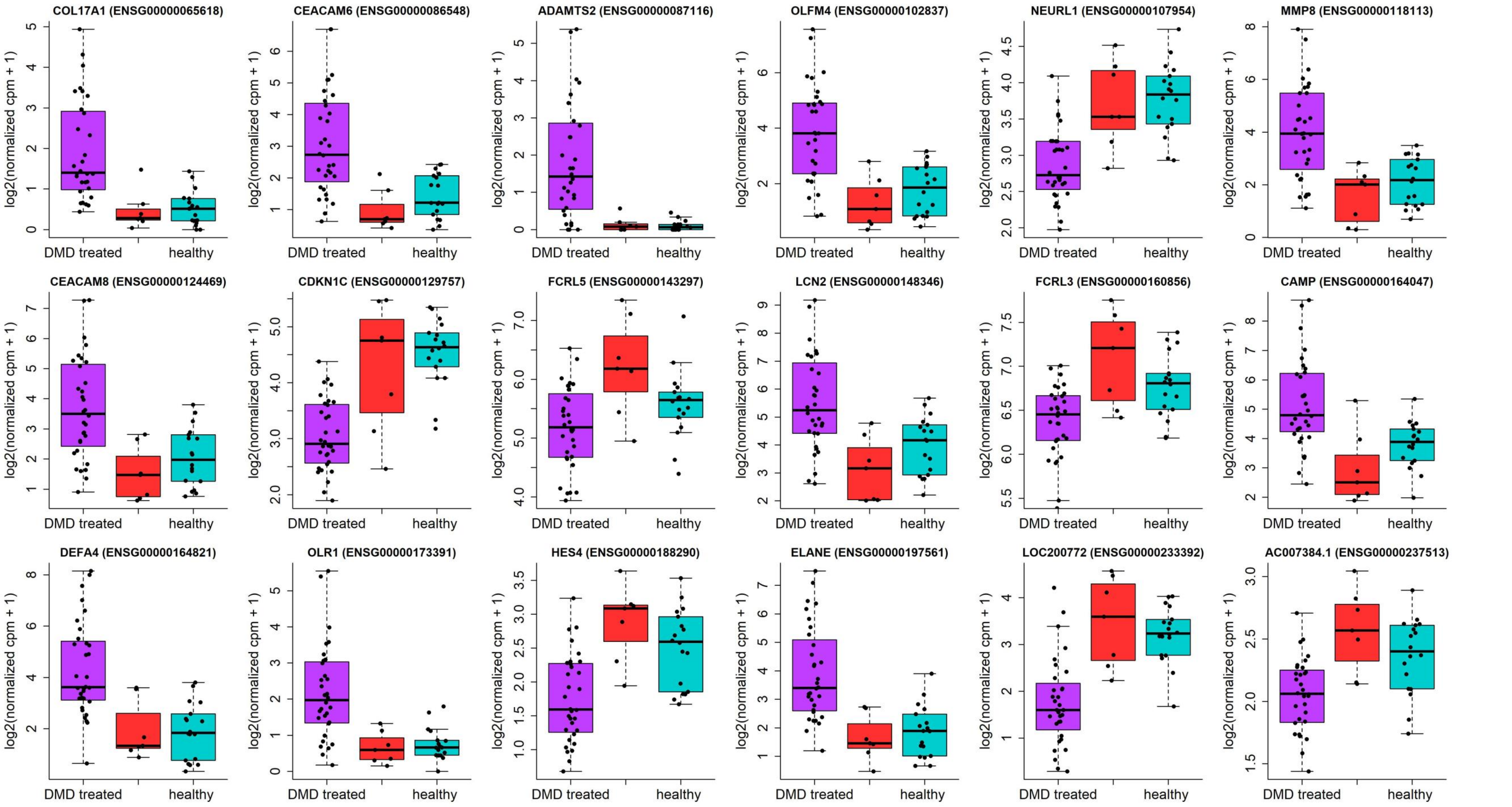

Appendix Figure S4

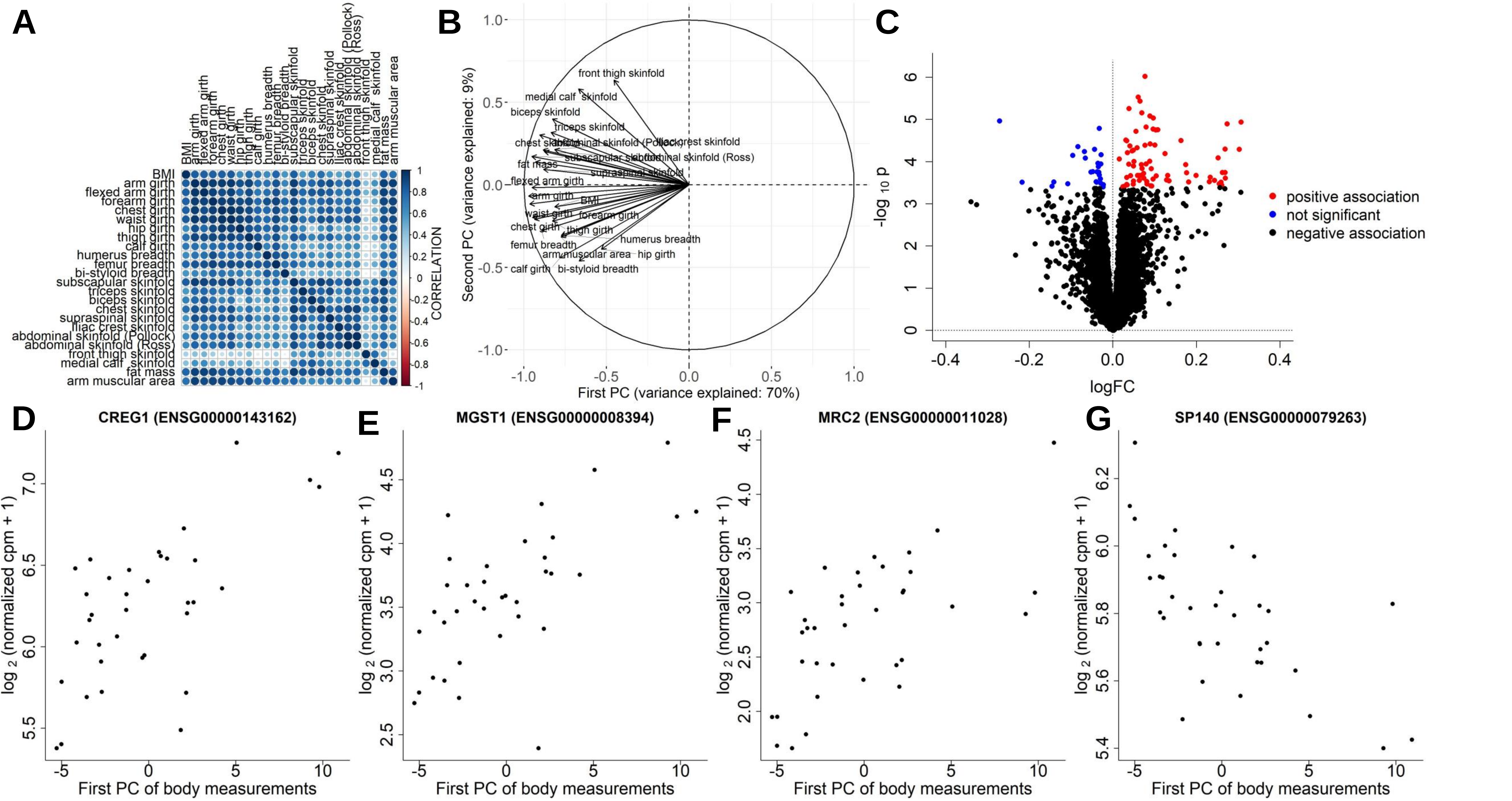

Appendix Figure S5

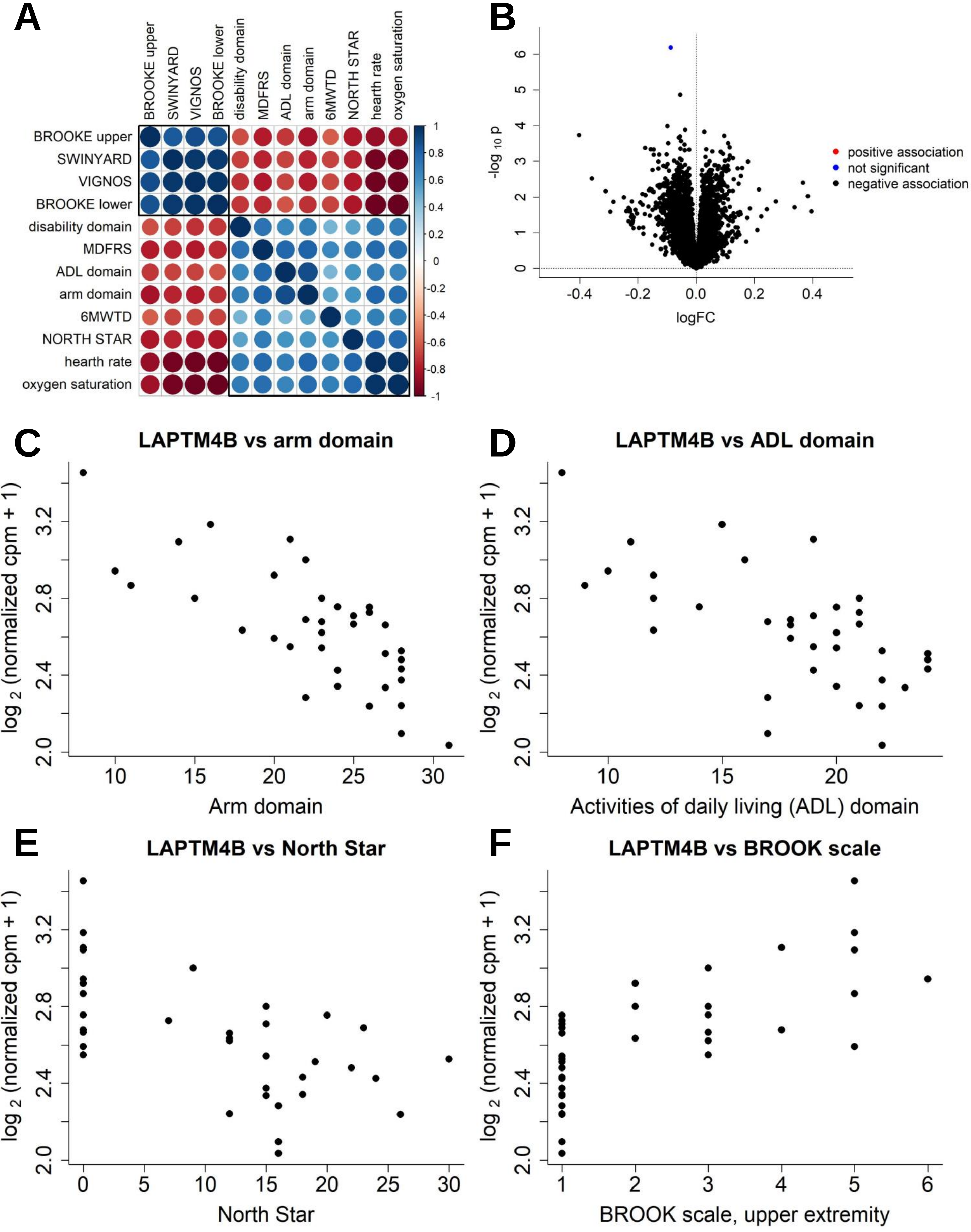

Appendix Figure S6
